# Machine Learning-Based Ear Thermal Imaging for Emotion Sensing

**DOI:** 10.3390/s26041248

**Published:** 2026-02-14

**Authors:** Budu Tang, Wataru Sato

**Affiliations:** 1 Graduate School of Informatics, Kyoto University, Yoshida-Honmachi, Sakyo, Kyoto 606-8507, Japan; tang.budu.53s@st.kyoto-u.ac.jp; 2 Psychological Process Research Team, Guardian Robot Project, RIKEN, 2-2-2 Hikaridai, Seika-cho, Soraku-gun, Kyoto 619-0288, Japan

**Keywords:** emotional arousal, ear thermal imaging, arousal and valence, machine learning, pixel-level analysis

## Abstract

Thermal imaging, which is contact-free, light-independent, and effective in detecting skin temperature changes that reflect autonomic nervous system activity, is expected to be useful for emotion sensing. A recent thermography study demonstrated a linear relationship between ear temperatures and emotional arousal ratings. However, whether and how ear thermal changes may be nonlinearly related to subjective emotions remains untested. To address this issue, we reanalyzed a dataset that included ear thermal images and self-reported arousal ratings obtained while participants watched emotion-eliciting films. We employed linear regression and two nonlinear machine learning models: a random forest model and a ResNet-50 convolutional neural network. Model evaluation using mean squared error and correlation coefficients between actual arousal ratings and model predictions indicated that both machine learning models outperformed linear regression and that the ResNet-50 model outperformed the random forest model. Interpretation of the ResNet-50 model using Gradient-weighted Class Activation Mapping and Shapley additive explanation methods revealed nonlinear associations between temperature changes in specific ear regions and subjective arousal ratings. These findings imply that ear thermal imaging combined with machine learning, particularly deep learning, holds promise for emotion sensing.

## 1. Introduction

Infrared thermography is a contact-free, light-independent method for monitoring emotion-related physiology [1,2,3]. Because thermal cameras measure emitted rather than reflected radiation, they remain effective even in environments in which visible-light systems perform poorly [2,3]. Moreover, activation of the sympathetic nervous system induces vasoconstriction in cutaneous blood vessels and lowers skin temperature, allowing frame-level thermal fluctuations to serve as an immediate proxy for underlying autonomic changes [4]. Thermal imaging requires no physical contact and avoids disturbing participants, making it especially suitable for monitoring vulnerable populations such as infants and clinical patients [5,6]. In addition, recent advances in low-cost uncooled microbolometer arrays and on-board calibration have improved spatial and temporal resolution, enabling thermography to be applied in large-scale, real-world emotion-sensing scenarios [7]. Several previous studies have examined full-face thermal imaging during emotional states and reported that the temperatures of certain facial regions, including the nose tip, are negatively associated with subjective arousal states [8,9,10,11,12].

A recent study further measured thermal images from the ear for emotion sensing [13]. Compared to facial infrared thermography, ear thermography offers unique advantages in contexts in which face masks are worn, such as during influenza seasons or in medical settings. Anatomical and physiological studies have revealed dense sympathetic and parasympathetic innervation in several subregions of the ear, implying its potential to exhibit emotion-related vascular changes [14]. In that study [13], the researchers conducted an experiment in which unilateral (right) ear thermal images were acquired while participants viewed emotionally evocative video clips, along with second-by-second arousal ratings. Linear associations between ear temperature changes and subjective ratings were analyzed using pixel-wise statistical parametric mapping analysis [15]. The results demonstrated robust negative associations between temperature in certain posterolateral ear regions and subjective arousal. These findings imply that ear thermal imaging may serve as a reliable index of subjective emotional arousal.

However, whether and how ear thermal changes are nonlinearly related to subjective emotional states remain untested. Machine learning (ML) methods offer a powerful framework for uncovering nonlinear associations in high-dimensional image data [16]. A previous study in facial thermal imaging has demonstrated that ML models outperform linear models in estimating emotional arousal [17], implying their broader applicability to peripheral thermal signals. In the literature on estimating emotional responses from non-thermal facial images using ML approaches, several studies have reported that deep learning-based models outperformed conventional ML models [18,19,20]. Based on these findings, we hypothesized that ML models, particularly deep learning-based models, could more accurately estimate subjective emotional responses from thermal ear data than traditional linear regression models. If substantiated, such advancements could refine emotion estimation methodologies and open new avenues for real-world applications, including mental health assessment and consumer behavior modeling.

The superior predictive accuracy of ML models comes at a cost: their internal computations are largely opaque, limiting researchers’ ability to determine why a particular thermal pattern yields a specific arousal estimate. This “black-box” property constrains scientific interpretability and can undermine trust in clinical or consumer-facing applications [21]. To gain insight into the mechanisms underlying ML-based arousal prediction from ear thermal images, a two-stage interpretability analysis can be used that combines spatial attention visualization with feature contribution analysis. First, Gradient-weighted Class Activation Mapping (Grad-CAM) identifies the anatomical regions of the ear that are most influential for arousal prediction by generating coarse localization maps from gradients in the final convolutional layer [22]. Second, Shapley additive explanations (SHAP), a game-theoretic method that attributes additive contributions from each input pixel to the final prediction [23], can be used to elucidate further the model’s internal decision processes. Whereas Grad-CAM indicates where the model attends spatially, SHAP quantifies how local thermal features increase or decrease the predicted arousal relative to a baseline. We hypothesized that these analyses would reveal physiologically plausible auricular subregions (e.g., the helix rim) whose temperature dynamics disproportionately influence model outputs, thereby providing evidence that the network relies on meaningful physiological cues.

To evaluate these hypotheses empirically, we reanalyzed data from a previous study that recorded right-ear infrared videos while participants viewed short, emotion-eliciting film excerpts [15]. Following the initial viewing and thermal data acquisition, each excerpt was replayed twice. During these replays, participants used a slider-type affect rating dial [24] to retrospectively and dynamically rate valence or arousal. Time-aligned thermal frames and self-report traces were then paired and used to train three arousal-prediction models: a multiple linear regression model using posterolateral ear regions as the regions of interest (ROIs) as a baseline, a random forest (RF) model as a representative conventional ML approach [25], and a ResNet-50 convolutional neural network as a representative deep learning-based ML model [26]. The predictive performance of each model was evaluated using a leave-one-participant-out cross-validation (LOPOCV) scheme. For interpretability analyses, we focused on the ResNet-50 model, which outperformed the other two models, and applied Grad-CAM to derive spatial importance maps. Subsequently, SHAP analysis was conducted to quantify how local ear temperature variations within these subregions influenced the model’s moment-to-moment arousal predictions.

## 2. Materials and Methods

### 2.1. Participants

The study cohort comprised 14 healthy adults from Japan (mean ± *SD* age = 22.0 ± 2.6 years; 7 females, 7 males). The required sample size was determined using an *a priori* power analysis conducted with G*Power (version 3.1.9.2) [27]. We assumed a one-tailed *t*-test with an α level of 0.05 and statistical power of 0.80. The effect size was assumed to be *d* = 0.89, derived from a previous study comparing emotion-sensing performance between ML models and linear regression models using different physiological signals (i.e., facial electromyography) [20]. This analysis indicated that a minimum of 10 participants would be required. Recruitment was conducted via advertisements at Kyoto University, and participants were compensated with a 4000 JPY book coupon for their participation. The inclusion criteria required that all participants be native Japanese speakers with no self-reported history of psychiatric or neurological disorders and with normal or corrected-to-normal vision. In addition, participants were required to be naïve to the experimental stimuli and to provide consent for both physiological and subjective measurements. The study protocol was approved by the RIKEN Ethics Committee, and all procedures were conducted in accordance with the Declaration of Helsinki and institutional guidelines. Written informed consent was obtained from all participants prior to participation, following a detailed explanation of the experimental procedures. The results of the linear analysis based on the present dataset have been reported previously [13].

### 2.2. Apparatus

Stimulus presentation was controlled using Presentation software (Neurobehavioral Systems, Berkeley, CA, USA) running on a Windows-based workstation (HP Z200 SFF, Hewlett-Packard Japan, Tokyo). Visual stimuli were displayed on a 19-inch monitor (HM903D-A; Iiyama, Tokyo, Japan) with a resolution of 1024 × 768 pixels. Continuous self-reports were collected using an optical mouse (MS116, Dell, Round Rock, TX, USA), which transmitted responses to a separate laptop operating on Windows (CF-SV8; Panasonic, Tokyo, Japan). Ear-temperature recordings were acquired using an infrared camera (FLIR A655sc, FLIR Systems, Wilsonville, OR, USA; 640 × 480 pixels, 50 Hz) controlled by Research IR Max v. 4.40 software. The camera was positioned approximately 0.77 m to the participant’s right side and aligned with the ear. Although full-face RGB and thermal videos were recorded concurrently, these data were not included in the present analyses.

### 2.3. Stimuli

**Figure 1:**
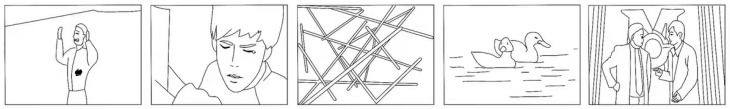
Illustrations of representative frames of the five emotion-eliciting film excerpts used as stimuli. From left to right: anger, sadness, neutral, contentment, and amusement conditions.

To elicit distinct affective states, five film excerpts were selected whose emotional effectiveness for Japanese audiences has been validated previously [10]. Representative frames from the five excerpts are presented in Figure 1 to illustrate the stimuli used for each emotion condition. The clips were intended to induce anger (Cry Freedom), sadness (The Champ), neutrality (Abstract Shapes), contentment (Wild Birds of Japan), and amusement (M-1 Grand Prix The Best 2007–2009). The mean ± *SD* duration of the clips was 175.8 ± 22.2 s, with individual lengths of 157 s, 172 s, 206 s, 148 s, and 196 s, respectively. All stimuli were presented at a resolution of 640 × 480 pixels, subtending approximately 25.5° horizontally and 11.0° vertically. Prior to the main experimental session, two brief practice clips (The Silence of the Lambs and the Color Bars) were shown to familiarize participants with the task.

### 2.4. Procedure

Experimental sessions were conducted in a sound-attenuated and electrically shielded booth maintained at an ambient temperature between 23.5 °C and 24.5 °C, as monitored using a TR-76Ui thermometer (T&D Corp., Matsumoto, Japan) to minimize potential effects of minor environmental temperature fluctuations on skin temperature. Upon arrival, participants received a detailed explanation of the experiment, provided written informed consent, and remained seated quietly for approximately 10 min to acclimate to the room environment to allow participants to settle and reach a stable physiological state before thermal recording. Prior to the main experimental session, two brief practice clips were presented to familiarize participants with the task.

Participants viewed the film clips from a distance of 0.77 m while ear temperature was continuously recorded by the infrared camera. Each trial began with a 1 s fixation cross, followed by a 10 s white screen to establish a baseline. After the completion of each clip, a second 10 s white screen served as a post-stimulus baseline. Participants then rated overall valence and arousal using a nine-point affect grid by pressing the corresponding number keys. They were instructed to attend closely to the stimuli and to withhold their ratings until the clip had ended. The five film excerpts were presented in a pseudorandom order across participants to mitigate possible order effects. Inter-trial intervals consisted of a black screen lasting 24–30 s, with durations randomized across trials to reduce carry-over effects between clips. Digital trigger signals indicating stimulus onset were recorded concurrently with the thermal data.

Subsequently, the same five film excerpts were presented again during a cued recall session. Participants reconstructed their moment-to-moment emotional experiences from the initial viewing by moving a mouse, with the x- and y-coordinates sampled at 10 Hz. Valence ratings were obtained first, followed by arousal ratings, yielding continuous emotional time courses. Dynamic ratings were collected during stimulus replay (cued recall) rather than during the initial viewing to minimize movement and attentional interference during thermal recording. Cued recall ratings have been shown to approximate real-time emotional responses to film stimuli closely [28,29].

### 2.5. Workflow

**Figure 2:**
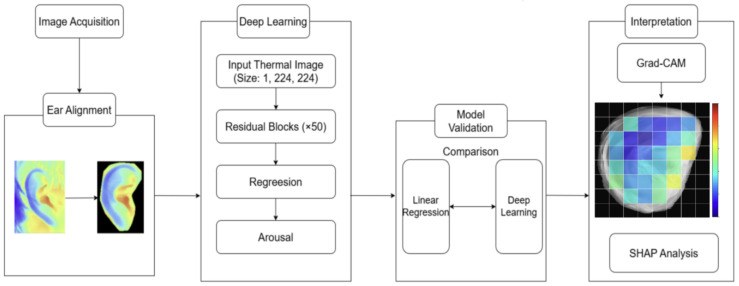
Workflow for analyzing emotional responses using ear thermal imaging. Grad-CAM, Gradient-weighted Class Activation Mapping; SHAP, Shapley additive explanations.

The overall workflow of the study is summarized in Figure 2. The process began with the acquisition of raw ear thermal video images, followed by a preprocessing step involving ear alignment, during which each frame was rotated, translated, and scaled to isolate and position the external ear in a standardized orientation for analysis. The aligned 224 × 224 thermal maps were then provided as input to the deep learning stage. A ResNet-50 regression model was used, which processed the single-channel ear images through 50 residual blocks and output a continuous arousal estimate for each frame. For model validation, the predictive performance of this deep learning model was evaluated using a LOPOCV scheme and compared with that of a baseline ordinary least-squares linear regression model. In the final interpretation stage, Grad-CAM was first applied to generate spatial importance maps across the ear, which were then summarized on an 8 × 8-grid. Based on these maps, the three grid regions with the highest contributions were selected, and SHAP analysis was subsequently performed for each region to quantify how local ear temperature within these subregions contributed to the model’s arousal predictions.

### 2.6. Preprocessing

**Figure 3:**
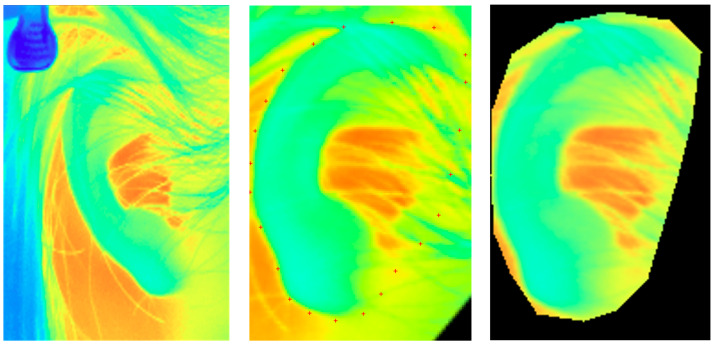
The ear image preprocessing pipeline. The left panel shows a raw thermal frame. The center panel displays the image after rotational alignment and scaling, with the manual annotation points (red crosses) used to define the ear boundary for segmentation mask generation. The right panel presents the final segmented ear image, precisely isolated from the background and used as input to the deep learning model.

A two-stage preprocessing pipeline was developed to segment and normalize the ear region in each frame of the raw thermal video accurately. This procedure is visually summarized in Figure 3. The first stage focused on global alignment and scaling. In the initial frame of each video, an operator manually annotated two key points corresponding to the superior and inferior extremities of the auricle. These key points were then automatically tracked across all subsequent frames using a custom algorithm. The coordinates of the tracked points were used to apply rotational and scaling transformations, thereby standardizing the orientation and size of the ear across the entire image sequence, thereby reducing variability due to small head/postural movements across frames.

Following this global alignment, a more precise segmentation procedure was applied. This step began with manual annotation of multiple points delineating the perimeter of the ear in the initial frame. These points were used to generate an accurate segmentation mask. A tracking algorithm then propagated this mask across subsequent frames, allowing minor deformations to accommodate subtle ear movements, and the resulting mask for each frame was saved. To ensure segmentation accuracy, the pipeline incorporated a semi-automated quality control process. When an observer detected a tracking failure in a given frame, one of two corrective actions could be taken: reverting to a previously validated mask from an earlier frame and restarting the tracking process from that point, or manually redrawing the mask on the problematic frame to establish a new reference for subsequent tracking. This robust preprocessing procedure ensured that a highly accurate segmented ear image was obtained for each frame, rendering the data suitable for input into the deep learning model.

### 2.7. Model Training

To predict emotional arousal from auricular thermal patterns, we trained and validated three regression models spanning a range of complexity, from a simple linear baseline to a deep convolutional network.

As a baseline approach, we implemented an ordinary least-squares linear regression model. Single-channel preprocessed ear images were first resized to 64 × 64 pixels, yielding a two-dimensional array of thermal intensity values. Each frame was then flattened into a 4096-dimensional feature vector, which served as the direct input to the linear regression model for mapping pixel intensities to continuous arousal scores. The data used for this analysis were extracted from the three ear ROIs exhibiting the strongest linear associations with arousal ratings in the previous statistical parametric mapping analysis [13], and linear regression models were fitted using the average temperature values within these regions.

Next, we trained a RF regression model to capture potential nonlinear relationships while maintaining a relatively interpretable structure. For this model, aligned ear images were resized to 224 × 224 pixels and converted into flattened feature vectors. These vectors were used to train an RF regressor with 300 trees, a maximum tree depth of 15, and a minimum leaf size of 2 to predict arousal values.

Finally, the primary deep learning model was based on a ResNet-50 architecture fine-tuned end to end for regression. The network was initialized with ImageNet-pretrained weights, and the original classification head was replaced with a single linear output node to generate a continuous arousal estimate. Input ear images were converted to single-channel thermal maps, resized to 224 × 224 pixels, and normalized before being fed into the network. Model training utilized the Adam optimizer (learning rate = 1 × 10^−4^, weight decay = 1 × 10^−4^), a batch size of 32, and mean squared error (MSE) as the loss function over 10 epochs.

To evaluate model generalization, LOPOCV was applied to all three models. In each fold, data from one participant were reserved as the test set, while data from the remaining participants were used for training. This procedure ensured that performance estimates reflected the ability of the models to predict arousal in previously unseen individuals. Model performance was quantified using MSE and the Pearson correlation coefficient between predicted and self-reported arousal trajectories, enabling direct comparison across the linear regression, RF, and ResNet-50 models.

### 2.8. Statistical Analysis

To compare the predictive accuracy of the three regression models (linear regression, RF, and ResNet-50) formally, statistical analyses were conducted on the performance metrics derived from the LOPOCV procedure. The LOPOCV scheme yielded outcome measures for each participant, consisting of an MSE value and a Pearson correlation coefficient for each model. To assess whether statistically significant differences existed between models, pairwise one-tailed *t*-tests were performed.

Preplanned contrasts using one-tailed *t*-tests were conducted for both MSE values and Pearson correlation coefficients to evaluate the following comparisons: ResNet-50 versus linear regression, RF versus linear regression, and ResNet-50 versus RF. The significance threshold (α) for all statistical tests was set at 0.05.

### 2.9. Model Interpretation Methods

To elucidate the predictive mechanisms underlying the deep learning model, a two-stage interpretability analysis was performed on the ResNet-50 model, combining spatial attention visualization with feature contribution analysis.

First, to identify the anatomical regions of the ear that were most influential for arousal prediction, Grad-CAM was applied. Grad-CAM leverages the gradients of the target output with respect to the feature maps in the final convolutional layer to generate coarse localization maps that highlight regions of importance within the input image. For quantitative analysis, the standardized ear mask was partitioned into an 8 × 8 grid. Grad-CAM intensities were computed for each frame, upsampled to the input resolution, and aggregated within each grid cell to produce a normalized group-level importance map.

Second, to characterize the specific relationship between local ear temperature and the model’s output, SHAP was used. Whereas Grad-CAM indicates where the model attends, SHAP quantifies how individual feature values drive predictions higher or lower relative to a baseline. Using the same 8 × 8 grid representation, the grid cells exhibiting the highest contributions were identified. For these high-importance ROIs, mean temperature values and corresponding SHAP values were extracted for every frame across all participants. These relationships were then quantified by fitting first- through fourth-degree polynomial regression models to reveal potential nonlinear patterns. We evaluated the models using adjusted R^2^, root mean squared error (RMSE), and Bayesian information criterion (BIC).

## 3. Results

### 3.1. Subjective Ratings

**Figure 4:**
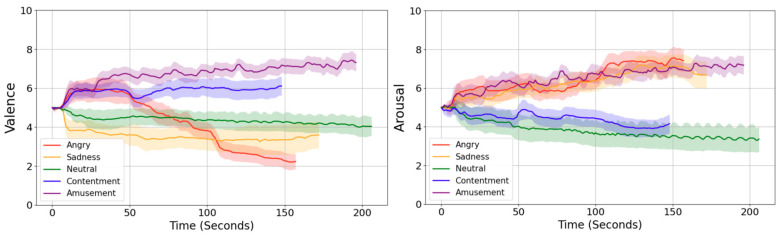
Mean moment-to-moment valence (**left**) and arousal (**right**) ratings evoked by the five emotion-eliciting film clips. The traces illustrate how self-reported affect evolved over time, revealing clip-specific differences in both valence and arousal dynamics. Shaded areas represent the 95% confidence intervals across participants.

Figure 4 presents the averaged, second-by-second trajectories of valence (left panel) and arousal (right panel) across all participants. The time courses indicate that the emotion-inducing film excerpts elicited clear and dynamic changes in both affective dimensions, with each clip producing a distinct and characteristic pattern of temporal variation.

### 3.2. Estimation Performance

**Figure 5:**
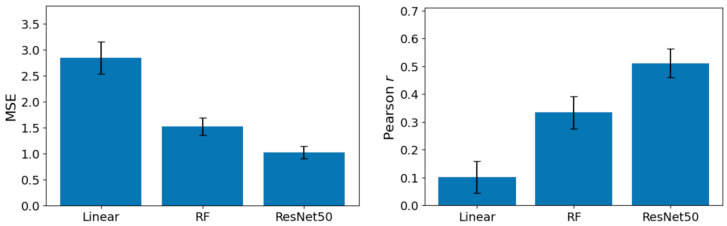
Mean ± standard error of mean squared error (MSE; **left**) and Pearson correlation coefficients (**right**) for the linear regression, random forest (RF), and ResNet-50 models predicting subjective arousal ratings.

Figure 5 summarizes the across-participant performance of the three arousal-prediction models evaluated using the LOPOCV procedure. The left panel displays mean ± standard error values for MSE, whereas the right panel shows mean ± standard error values for the Pearson correlation coefficient (*r*) between model predictions and self-reported arousal ratings. Across both performance metrics, the ResNet-50 model achieved the lowest MSE and the highest correlation, the RF model showed intermediate performance, and the linear regression model exhibited the highest MSE and the lowest correlation. Error bars represent the standard error of the mean across participants.

Pairwise one-tailed *t*-tests revealed that, relative to the linear regression model, the ResNet-50 model demonstrated significantly lower MSE (*t*(13) = 5.68, *p* < 0.001, *d* = 1.52) and higher Pearson correlation coefficients (*t*(13) = 5.30, *p* < 0.001, *d* = 1.42). The RF model also outperformed the linear model, exhibiting lower MSE (*t*(13) = 3.83, *p* = 0.001, *d* = 1.02) and higher correlation (*t*(13) = 2.66, *p* = 0.009, *d* = 0.71). Furthermore, the ResNet-50 model achieved significantly lower MSE (*t*(13) = 2.38, *p* = 0.017, *d* = 0.64) and higher Pearson correlation coefficients (*t*(13) = 2.09, *p* = 0.033, *d* = 0.56) compared to the RF model. Collectively, these results indicate that both ML models outperformed the linear regression model, with the ResNet-50 model demonstrating superior predictive performance relative to the RF model.

### 3.3. Model Interpretation

**Figure 6:**
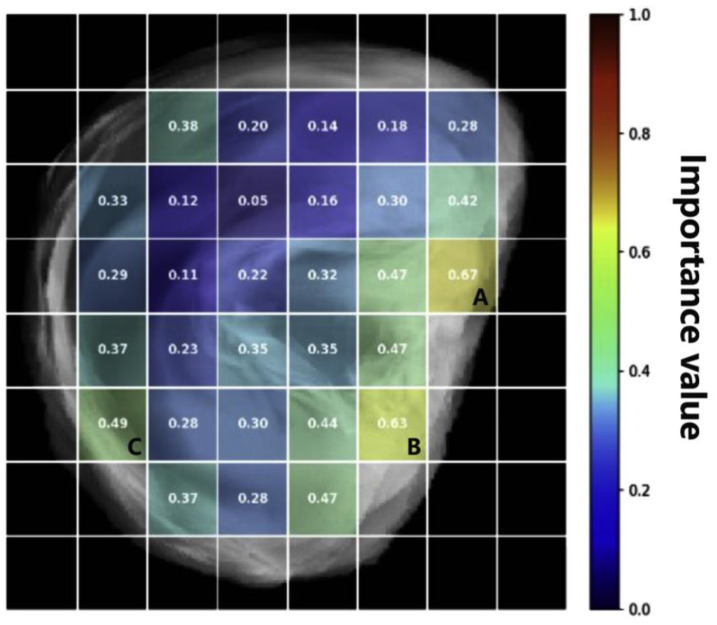
Group-level Gradient-weighted Class Activation Mapping contribution map for the ear, with an 8 × 8 grid overlaid on a template ear image showing normalized (0–1) ResNet-50 importance values for each auricular subregion. Alphabet letters indicate the regions of interest for subsequent Shapley Additive Explanations analyses (A: superior preauricular region; B: tragus; C: lower helix).

Grad-CAM was first applied to the ResNet-50 model, which outperformed the other two models, to visualize which auricular regions were most influential for arousal prediction. Grad-CAM is a gradient-based visualization technique that uses the derivatives of the model output with respect to feature maps in a convolutional layer to generate a coarse importance map over the input image. For each frame, Grad-CAM was computed at a late convolutional layer, and the resulting map was upsampled to the input resolution. The spatial distribution of model attention was then quantified by partitioning the ear mask into an inner 8 × 8 grid. Within each grid cell, mean Grad-CAM intensity values were calculated, averaged within each participant, and subsequently combined across participants using a weighted mean. The resulting group-level map was normalized to a range of 0–1 within the ear mask, such that larger values indicated stronger contributions of the corresponding subregions to the arousal prediction. The highest normalized Grad-CAM values were concentrated over the cymba concha and cavum concha, as well as along the helical rim, whereas several superior–posterior sectors of the ear exhibited comparatively low contribution values (Figure 6).

**Figure 7:**
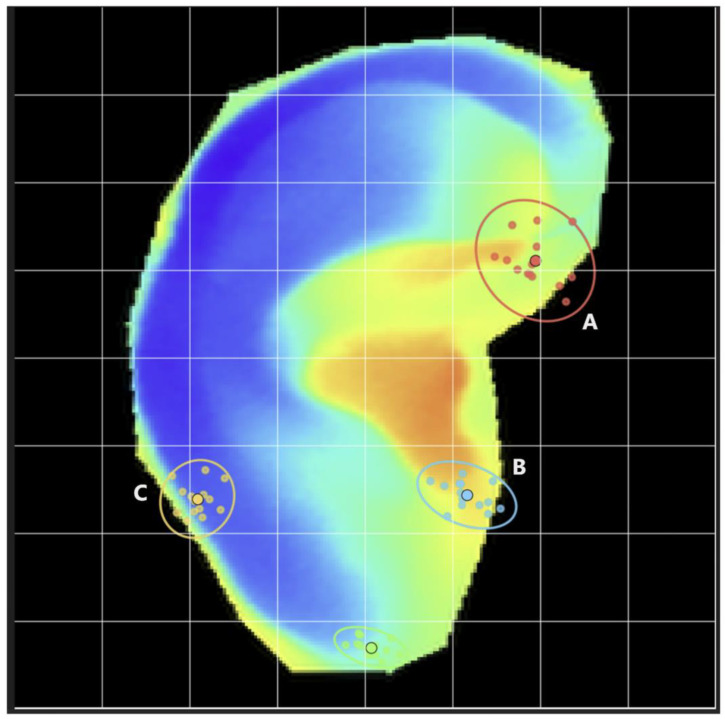
Cross-participant distributions of landmark locations in the normalized ear coordinate space. The background shows an example normalized ear thermal map with the overlaid 8 × 8 grid used for Grad-CAM and SHAP summarization. Colored dots indicate landmark locations from individual participants (*n* = 14) for Region A (superior preauricular region), Region B (tragus), Region C (lower helix), and the lobule. Larger markers indicate mean landmark locations, and ellipses depict the corresponding 95% dispersion in the normalized coordinate space.

To examine whether the 8 × 8 grid-based summarization yields consistent localization across participants after spatial normalization, we assessed the spatial dispersion of four landmarks in the normalized coordinate space (Figure 7). Three landmarks corresponded to the high-contribution regions highlighted in Figure 5 and were used for the subsequent SHAP analyses—Region A (superior preauricular region), Region B (tragus), and Region C (lower helix). Additionally, a fourth landmark was placed at the lobule as a clearly identifiable inferior reference point. Landmark coordinates were analyzed in the common normalized space, with a grid-cell width of 28 pixels. Across landmarks, the standard deviations along each axis ranged from 2.66 to 7.90 pixels, and the 95% dispersion ellipse semi-axes ranged from 5.76 to 20.94 pixels. Using the radial distance from each landmark to its mean location, all points were within 28 pixels. For Region A, the mean radial distance was 9.49 pixels with a 95th percentile of 16.68 pixels and a maximum of 17.35 pixels, whereas for the remaining 3 three landmarks, the 95th percentiles were below 12 pixels and the maxima were below 13 pixels.

To characterize further how local ear temperature relates to model attribution in the most influential subregions, the Grad-CAM analysis was complemented with SHAP. SHAP is a post hoc attribution method that assigns each input feature a contribution value reflecting how much it increases or decreases the model output relative to a baseline prediction. Using the same 8 × 8 grid representation, group-level SHAP maps were computed, and the three grid cells with the largest mean absolute SHAP values were identified: row 4, column 7 (Region A: superior preauricular region), row 6, column 6 (Region B: tragus), and row 6, column 2 (Region C: lower helix). For each of these ROIs, the mean temperature within the corresponding grid cell and the associated SHAP value were extracted for every frame and participant.

**Figure 8:**
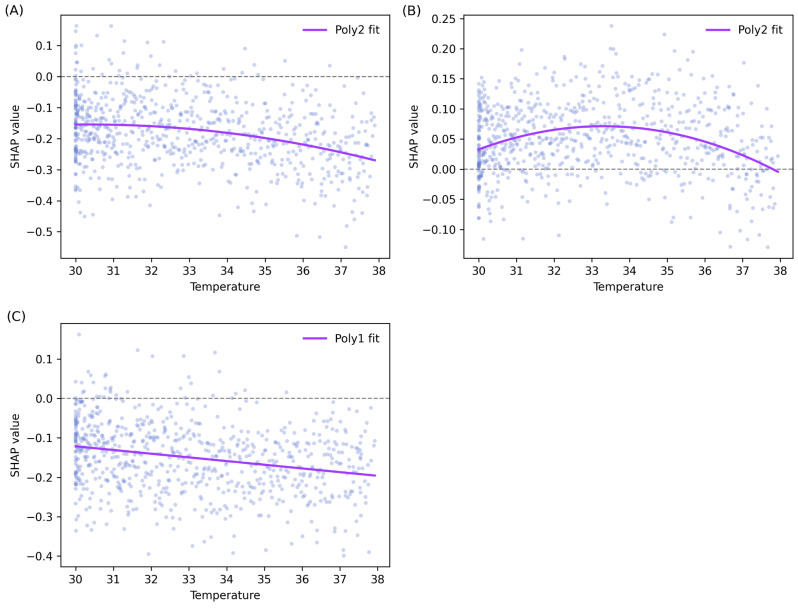
Scatterplots and optimal polynomial regression lines of Shapley Additive Explanations (SHAP) values for the three high-contribution auricular regions ((**A**) superior preauricular region; (**B**) tragus; (**C**) lower helix), illustrating the nonlinear and linear relationships between local ear temperature and model attribution within these subregions.

To quantify these temperature-attribution relationships systematically, first- through fourth-degree polynomial regression models were fitted to the temperature-SHAP data for each region and compared, based primarily on the BIC (Table 1). The relationships are summarized in Figure 8, which overlays the selected polynomial fits on the frame-level scatterplots. Regions A and B are best described by second-degree polynomial models, while Region C is best described by a first-degree polynomial model. Specifically, Regions A (the superior preauricular region) and B (the tragus) exhibited an inverted-U-shaped relationship, with slightly higher SHAP values at intermediate temperatures, while Region C (lower helix) showed an almost monotonic decrease in SHAP values with increasing temperature.

## 4. Discussion

Our evaluation of model predictive accuracy using the LOPOCV scheme demonstrated that both ML models—the RF and ResNet-50 models—outperformed the linear regression model in predicting emotional arousal dynamics based on ear thermal imaging data. Although a previous study on ear thermography for emotion sensing reported primarily linear relationships between ear temperature and subjective arousal ratings [13], the present findings imply that ear temperature-arousal associations are not exclusively linear but also encompass nonlinear components. These results are consistent with earlier research showing that ML models outperform linear models in estimating subjective emotional states from frontal facial thermal images [12]. Moreover, our results indicate that the ResNet-50 model achieved superior predictive performance compared to the RF model when applied to ear thermal images. This pattern aligns with previous studies using non-thermal facial imaging, which reported that deep learning-based approaches outperformed conventional ML models in emotion sensing tasks [18,19,20]. Altogether, the present findings provide new evidence that ML models, particularly deep learning-based architectures, can more effectively estimate subjective emotional arousal from ear thermal images than traditional linear regression models.

To interpret further the behavior of the ResNet-50 model in arousal prediction, we visualized its spatial attention patterns using Grad-CAM. For each frame, Grad-CAM maps were extracted from a late convolutional layer, upsampled to a standardized ear mask, and summarized on an 8 × 8 grid before aggregation within and across participants (Figure 3). At the group level, the model did not weight the auricle uniformly; instead, it assigned disproportionately high importance to specific subregions, including anteromedial areas (i.e., the superior preauricular region and the tragus) and a posterolateral area (i.e., the lower helix). In other words, the network relied on anatomically specific auricular subregions rather than treating the ear as a spatially homogeneous thermal source. This observation is consistent with anatomical and physiological evidence indicating that different auricular regions vary in tissue composition, vascular supply, heat-dissipation properties, and autonomic nervous system innervation [30,31]. Accordingly, our analysis identified auricular subregions whose vascular characteristics imply stronger modulation by emotion-related autonomic nervous system activity.

To assess whether the local thermal cues used by the model to estimate arousal can be quantitatively interpreted, we used SHAP to examine how the temperature of high-contribution auricular subregions influences moment-to-moment arousal predictions. Specifically, we combined Grad-CAM defined high-contribution regions with SHAP. For each frame, we extracted the mean temperature within that region and computed the SHAP value of this local temperature for the frame’s predicted arousal. Two primary findings emerged. First, the SHAP dependence curve in the anteromedial regions (i.e., the superior preauricular region and the tragus) showed negative quadratic associations between temperature and arousal ratings: local temperature increased while arousal feeling increased at a certain level. These results may be compatible with anatomical data indicating that the anteromedial regions receive innervation from the auricular branch of the vagus nerve (i.e., the primary nerve of the parasympathetic division of the autonomic nervous system) [32,33]. A previous physiological study investigated the relationship between pharmacologically manipulated parasympathetic activity and heart rate variability, a common measure of parasympathetic activity [34], and found a negative quadratic relationship [35]. Therefore, the inverted-U-shaped relationships between temperature changes and subjective arousal in these regions may reflect parasympathetic activity. Second, SHAP values in the posterolateral region (i.e., the lower helix) showed a linear, negative relationship between temperature and arousal ratings. These results were consistent with previous findings using linear analysis [13]. The results were also in agreement with anatomical and physiological data indicating that the posterolateral ear region receives blood supply primarily maintained by the posterior auricular artery and branches of the superficial temporal artery, governed by the sympathetic division of the autonomic nervous system [14]. In summary, these findings highlight the linear and nonlinear relationships between temperature and subjective emotional arousal in the ear subregions.

The present results highlight several advantages of ML-based ear thermal imaging for real-world emotion monitoring. In situations where the nose and cheeks are partially or fully covered, for example, during influenza seasons, pollen allergy periods, or in clinical environments requiring face masks, face-centered thermography may be unreliable, whereas the external ear typically remains visible and accessible for contact-free, continuous monitoring. In addition, the auricle is mechanically more stable than many facial regions, being less affected by speech, chewing, and expressive facial movements, which reduces motion-related artifacts and facilitates consistent ROI tracking over time. These properties render auricular thermography particularly suitable for unobtrusive assessment of anxiety or distress in clinical and counseling contexts, offering an objective physiological measure with minimal burden on the user. Even when full facial information is available, ear temperature dynamics can provide a complementary channel to face-focused analyses, supplying an independent index of arousal-related vasomotor activity and enhancing robustness through multi-site data integration.

Despite these promising findings, several limitations should be acknowledged. First, although the LOPOCV scheme indicated model generalizability, the sample size was relatively small (*n* = 14) and the participants were limited to young Japanese adults. This limited cohort may not have fully captured interindividual variability in ear temperature responses to emotional arousal and, hence, may constrain the generalizability of the present findings across age groups and cultural backgrounds. Additionally, the stimulus set comprised only five emotion-eliciting film excerpts, which may have limited the diversity of affective contexts and response dynamics represented in the data. Future studies should rigorously evaluate the robustness of these effects by including larger, more diverse samples with broader age ranges and cultural backgrounds, as well as a wider range of emotional films.

Second, dynamic valence and arousal trajectories were obtained using a cued recall procedure during stimulus replay rather than online reporting during the initial viewing. Although this approach reduces movement and divided-attention demands during thermal acquisition, retrospective ratings may be subject to memory reconstruction bias. Future studies should directly compare online and cued recall ratings (e.g., within-subject) and evaluate whether potential discrepancies influence model training and performance.

Third, the present work treated ear thermal imaging as a single-modality predictor. This unimodal design did not allow us to validate the ear thermal thermography by cross-checking against other physiological measures. Moreover, the incremental benefit of ear thermography relative to established multimodal emotion-sensing approaches, as well as its potential complementarity with other signals, remains undetermined. Future studies should therefore acquire ear thermography alongside electrodermal activity, cardiovascular indices, or neural signals, and test convergent validity and multimodal integration.

Fourth, auricular temperature may be influenced by unmeasured individual factors such as skin condition, peripheral vascular regulation, and postural adjustments unrelated to emotion. Future studies should record these measures to quantify their effects and separate emotion-related signals from confounds.

Finally, the data were collected under controlled laboratory conditions with relatively stable ambient temperatures. While such control reduces nuisance variability and facilitates model training and interpretation, it may not fully reflect thermal noise and environmental variability in unconstrained real-world settings. Future studies should therefore evaluate model performance under more naturalistic conditions and develop calibration and robustness strategies that explicitly account for environmental fluctuations and recording-related variability.

## 5. Conclusions

This study presents the first systematic framework for estimating dynamic emotional arousal directly from ear thermal images using ML approaches. The results demonstrate that ML models, particularly the deep learning-based ResNet-50 architecture, outperformed conventional linear regression in capturing the nonlinear mapping between auricular temperature and subjective emotional states. By applying model interpretability techniques, including Grad-CAM and SHAP, we showed that the model relied on physiologically meaningful auricular subregions, specifically the superior preauricular region, the tragus, and the lower helix, to generate arousal predictions. Moreover, the observed linear and nonlinear relationships between local ear temperature and arousal imply region-specific involvement of the sympathetic and parasympathetic divisions of the autonomic nervous system. Collectively, these findings establish ML-based ear thermal imaging as an efficient and informative approach to emotion sensing. This method offers a promising avenue for unobtrusive emotion monitoring in real-world settings, particularly in situations in which facial occlusion or privacy considerations limit the applicability of traditional face-centric techniques.

## Figures and Tables

**Table 1:** Fit indices (adjusted R^2^, root mean squared error [RMSE], and Bayesian information criterion [BIC]) for polynomial models of degrees 1–4 applied to each region of interest (ROI).

ROI	Degree	Adjusted R^2^	RMSE	BIC
A (the superior preauricular region)	1	0.07712	0.10560	−3327.42
	**2**	**0.08422**	**0.10512**	**−3327.55**
	3	0.08374	0.10508	−3321.55
	4	0.08502	0.10494	−3316.99
B (the tragus)	1	0.00091	0.05993	−4169.18
	**2**	**0.0838** **7**	**0.05735**	**−4227.98**
	3	0.08476	0.05726	−4223.10
	4	0.08367	0.05728	−4216.61
C (the lower helix)	**1**	**0.06171**	**0.08712**	**−3613.31**
	2	0.06510	0.08690	−3610.40
	3	0.06412	0.08689	−3604.02
	4	0.06291	0.08689	−3597.45

The optimal models are indicated in bold.

## Data Availability

Data statistically tested during this study are included in this published article and its Supplementary Information Files. The thermal images are not publicly available due to privacy or ethical restrictions.

## Supplementary Materials

The following supporting information can be downloaded at: https://www.mdpi.com/article/10.3390/s26041248/s1, Supplementary Table S1: Correlation coefficient and mean squared error value between the actual and predicted values for each model of each participant.
